# Sex-dependent different clinicopathological characterization of Epstein–Barr virus-associated gastric carcinoma: a large-scale study

**DOI:** 10.1007/s10120-023-01460-8

**Published:** 2024-01-11

**Authors:** Ji-Hyun Kim, Nayoung Kim, Du Hyun Song, Yonghoon Choi, Eun-Bi Jeon, Sihyun Kim, Yu Kyung Jun, Hyuk Yoon, Cheol Min Shin, Young Soo Park, Dong Ho Lee, Hyeon Jeong Oh, Hye Seung Lee, Young Suk Park, Sang-Hoon Ahn, Yun-Suhk Suh, Do Joong Park, Hyung Ho Kim, Ji-Won Kim, Jin Won Kim, Keun-Wook Lee, Won Chang, Ji Hoon Park, Yoon Jin Lee, Kyoung Ho Lee, Young Hoon Kim, Soyeon Ahn

**Affiliations:** 1 Departments of Internal Medicine, Seoul National University Bundang Hospital, 82, Gumi-Ro 173 Beon-Gil, Bundang-Gu, Seongnam, Gyeonggi-Do 13620 South Korea; 2 Departments of Pathology, Seoul National University Bundang Hospital, Seongnam, South Korea; 3 Departments of Pathology, Seoul National University College of Medicine, Seoul, South Korea; 4 Departments of Surgery, Seoul National University Bundang Hospital, Seongnam, South Korea; 5 Departments of Radiology, Seoul National University Bundang Hospital, Seongnam, South Korea; 6 Departments of Internal Medicine, Seoul National University College of Medicine, Seoul, South Korea; 7 Departments of Surgery, Seoul National University College of Medicine, Seoul, South Korea; 8 Departments of Radiology, Seoul National University College of Medicine, Seoul, South Korea; 9 Division of Statistics, Medical Research Collaborating Center, Seoul National University Bundang Hospital, Seongnam, South Korea

**Keywords:** Epstein–Barr virus, Gastric cancer, Survival, Lauren type, Sex

## Abstract

**Background:**

Epstein–Barr virus (EBV)-associated gastric cancer (EBVaGC) has been reported to account for approximately 5–16% of all GCs with good prognosis compared to EBV-negative GC. We evaluated the clinicopathological characteristics of EBVaGC including survival rate in South Korea.

**Methods:**

A total of 4,587 patients with GC who underwent EBV in situ hybridization (EBV–ISH) were prospectively enrolled at the Seoul National University Bundang Hospital from 2003 to 2021. Age, sex, smoking status, cancer type and stage, tumor size and location, histological type, molecular features and survival information were analyzed.

**Results:**

A total of 456 patients with GC (9.9%) were positive for EBV. The EBVaGC group displayed a higher proportion of males (*P* < 0.001), a predominant presence in the proximal stomach (*P* < 0.001), a higher proportion of undifferentiated cancer (*P* < 0.001), and a lower cancer stage (*P* = 0.004) than the EBV-negative group. Cox multivariate analyses revealed age (hazard ratio [HR] = 1.025, *P* < 0.001), tumor size (HR = 1.109, *P* < 0.001), and cancer stage (stage2 HR = 4.761, *P* < 0.001; stage3 HR = 13.286, *P* < 0.001; stage4 HR = 42.528, *P* < 0.001) as significant risk factors for GC-specific mortality, whereas EBV positivity was inversely correlated (HR = 0.620, *P* = 0.022). Furthermore, the EBVaGC group displayed statistically significant survival advantages over the EBV-negative cancer group in terms of both overall (*P* = 0.021) and GC-specific survival (*P* = 0.007) on the Kaplan–Meier survival curve. However, this effect was evident only in males.

**Conclusions:**

EBVaGC patients showed better prognoses despite their association with proximal location and poorly differentiated histology in male, probably due to the difference in immunity between males and females.

## Introduction

Although aggressive endoscopic screening in South Korea has led to a dramatic increase in early detection and treatment success rates over the past few decades, gastric cancer (GC) remains one of the most prevalent cancers not only in South Korea but also throughout East Asia, and remains as a leading cause of death [1]. The Cancer Genome Atlas (TCGA) project, published in 2014, classified gastric adenocarcinoma into four subtypes through comprehensive molecular analysis: (1) Epstein–Barr virus (EBV)-associated gastric carcinoma (EBVaGC), (2) gastric carcinoma with microsatellite instability, (3) gastric carcinoma with chromosomal instability, and (4) genetically stable gastric carcinoma [2, 3].

The EBV is a double-stranded DNA virus belonging to the Herpes virus family. It proliferates in the epithelial cells of the oropharynx and is primarily transmitted through the saliva. Primary infections usually occur during childhood and often remain unnoticed without significant symptoms. Subsequently, a latent infection is observed in B lymphocytes. More than 90% of the adult population worldwide has been reported to display a positive serological response to the virus [4]. It was classified as a Group I carcinogen by the International Agency for Research on Cancer (IARC) in 1997 due to its role in these cancers. [5, 6].

The diagnosis of EBVaGC using in situ hybridization (ISH) of the GC tissue is the gold standard [7]. Since Burke et al. first reported EBVaGC in 1990 [8], several studies have been conducted on this topic. Recent reports state that EBVaGC is the most common EBV-associated malignancy, with an estimated 75,000 to 90,000 cases occurring worldwide annually. It constitutes approximately 10% of all GCs and is prevalent in Far East Asia, where the incidence of GC is high [9]. In South Korea, 5.6–13% of all GC cases are associated with EBV infection [10–12]. EBVaGC exhibits features that differentiate it from typical GC. Several studies have demonstrated that EBVaGC is more common in males and tends to affect younger patients. However, certain meta-analyses failed to show the significance of age in EBVaGC [13–17]. Moreover, smoking is a risk factor for EBVaGC, and compared to non-smokers, the incidence of EBVaGC is 2.4 times higher in current smokers and two-fold higher in former smokers [18]. Using the Lauren classification, certain studies have demonstrated that the intestinal type predominates, whereas other studies indicate a higher prevalence of the diffuse-type. Other studies have reported no correlation between EBV positivity and Lauren classification [14, 15, 19, 20]. In addition, according to the World Health Organization (WHO) histology, it has been frequently associated with the poorly differentiated type [11]. In terms of cancer location, EBVaGC is commonly found in the proximal part of the stomach [11, 14]. Certain studies have reported that it has little relationship with *Helicobacter pylori* [21]. Furthermore, EBVaGC has fewer lymph nodes and less vascular invasion, leading to a better prognosis [13, 16, 22–25]. This could be related to the fact that EBVaGC is an immunogenic tumor leading to an active host cell immune response [26–28].

GC has sex-specific characteristics, which are highlighted by a higher proportion of diffuse-type GC in females, whereas GC in males is primarily located in the antrum [29, 30]. Furthermore, certain studies suggested that the number of activated immune processes was higher in females in GC [31, 32]. Based on this background, we hypothesized that EBVaGC has unique characteristics compared to other types of GC, and that these characteristics could differ depending on the sex. However, most previous studies had sample sizes of fewer than a few hundred study groups, and meta-analyses have often revealed conflicting results. Therefore, we conducted this study to obtain a more detailed understanding of the characteristics and prognosis of EBVaGC and investigate the different effects of EBV positivity depending on the sex in large-scale prospective long-term follow-up study at a single institution.

## Materials and methods

### Study population

Among 14,613 patients diagnosed with GC at the Seoul National University Bundang Hospital from May 2003 to February 2021 who were prospectively enrolled, 4587 patients who underwent EBV in situ hybridization (EBV–ISH) were found by reviewing electronic medical records (EMR). Medical records, including age, sex, smoking, tumor size and location, histologic type (according to the WHO and Lauren classification), molecular features (p53 expression and microsatellite instability [MSI]), cancer type and stage (according to the American Joint Committee on Cancer [AJCC] 8th edition), presence of lymphatic and vascular invasion, treatment methods, EBV positivity, and survival information, including causes of death, were collected and analyzed largely from surgical and medical cohorts established in 2003. This study was reviewed and approved by the Institutional Review Board (IRB) of SNUBH (IRB number B-2006-618-004). This study was performed following the protocols approved by the ethics committee.

### EBV–ISH

**Fig. 1:**
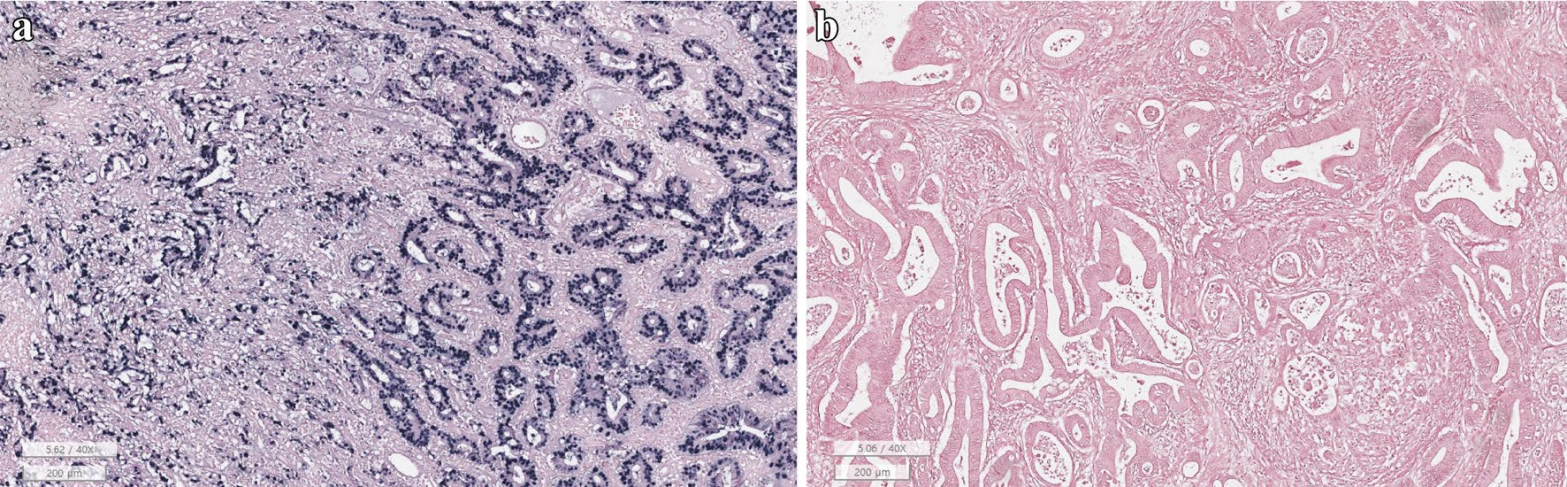
Image of Epstein-Barr virus (EBV) in situ hybridization (EBV-ISH) status. **a** An EBV-positive case shows strong nuclear positivity. **b** EBV-negative case in which nuclear staining of EBV-ISH was not detected

For the diagnostic criteria of EBVaGC, “EBV-positive” was referred to when tumor cells displayed positivity on EBV–ISH in the stomach specimens obtained via surgery or endoscopic treatments such as endoscopic mucosal dissection (ESD) (Fig. 1a). EBV-ISH were performed with a Probe: INFORM EBER (Epstein-Barr Virus Early RNA) Probe (material number- 05278660001), Detection kit: VENTANA ISH iVIEWBlue Detection Kit (material number-05278511001), ISH protease 2(material number-05273323001), Red stain II (material number-05272017001), all produced by Roche. The formalin fixed paraffin embedded (FFPE) tissues were cut into 3 μm-thick sections for EBV ISH. The sections were deparaffinized at 75 °C and pre-treated with ISH protease 2(material number-05273323001) for 8 min. Hybridization and visualization were done by pre-fixed protocol using probe and detection kit and counter stain was done with Red stain II for 4 min. In each case, a representative whole section slide containing the deepest invasive portion of the GC was selected for EBER in situ hybridization.

### Data variable

Tumor location was classified into three groups: upper, middle, and lower according to surgical pathological results or endoscopy. All tumors classified by the WHO classification system was re-categorized to differentiated (well and moderate), undifferentiated (poor and signet ring cell carcinoma), mixed, and other types of tumors. In addition, they were classified into intestinal and diffuse-types according to the Lauren classification system. They were confirmed after surgery or endoscopic treatment such as ESD. p53 positivity was defined as more than 10% staining of tumor cell nuclei for p53 immunohistochemistry. Early GC (EGC) is defined as a cancer invasion confined to the submucosa, whereas advanced GC (AGC) is defined as a cancer invasion extending beyond the submucosa. Cancer staging was applied based on the TNM stage according to the AJCC 8th edition. The dates and causes of death of study patients were cross-reviewed with data from the EMR and National Statistical Office for verification.

### Statistical analyses

Statistical analyses were performed using the SPSS software (version 27.0; IBM Corp., Armonk, NY, USA). Baseline characteristics and variables were analyzed using the chi-square and *t*-tests. The overall survival (OS) was defined as the time from the day of GC diagnosis to the day of death from any cause. In contrast, GC-specific survival (GCSS) was defined as the period from the day of GC diagnosis to the day of death, attributed specifically to GC. The follow-up period was up to 5 years, and if patients were lost to follow-up within 5 years, they were censored at their last follow-up date. OS and GCSS were estimated using the Kaplan–Meier method, and the differences between the curves were assessed using the log-rank test. Simultaneous multivariate adjustment of all covariates was performed using the Cox proportional hazards regression analyses to evaluate the significance of EBV positivity on survival. Variables with *P* value < 0.05 in the univariate analyses were used as covariates for multivariate analyses. A *P* value < 0.05 was considered significant.

## Results

### Clinicopathological features of patients according to the EBV status

Of the 4,587 patients who underwent EBV–ISH, 456 (9.9%) were positive for EBV, and 4131 (90.1%) were negative. The baseline characteristics are summarized in Table 1. The mean age of patients with EBVaGC was 61.2 years and of those in the EBV-negative group was 61.1 years without significance. EBV positivity was significantly more frequent in males (404, 88.6%) than females (52, 11.4%) (*P* < 0.001) and smokers (316, 69.8%) than in non-smokers (137, 30.2%) (*P* < 0.001). The tumor size was significantly smaller in the EBV-positive group than that in the EBV-negative group (mean: 3.6 cm vs. 3.9 cm, *P* = 0.030). The EBV-positive group predominantly had tumors in the upper third (236, 51.8%), whereas the EBV-negative group had a higher incidence in the lower third of the stomach (2415, 58.6%) (*P* < 0.001). Histologically, the EBV-positive group displayed undifferentiated type tumor (226, 49.5%), whereas the EBV-negative group more frequently showed the differentiated type (1770, 42.9%) (*P* < 0.001). According to the Lauren classification, the intestinal-type was present in 215 (49.8%) patients and the diffuse-type in 184 (42.6%) patients in the EBV-positive group, whereas the intestinal-type found in 2141 (54.7%) patients and the diffuse-type in 1631 (41.7%) patients in the EBV-negative group (*P* < 0.001). In the EBV-positive group, p53 mutations were less frequent than in the EBV-negative group (27, 12.2% vs. 825, 31.0%). Regarding the MSI, the EBV-positive group showed MSI-H (high) less than the EBV-negative group (2, 0.6% vs. 390, 10.7%). The EBV-positive group showed TNM stage 1 cancer more than the EBV-negative group (316, 69.3% vs. 2,505, 60.6%). Both lymphatic and vascular invasions were less frequent in the EBV-positive group compared to the EBV-negative group (lymphatic invasion: 104, 24.1% vs. 1405, 35.9%; vascular invasion: 30, 6.9% vs. 450, 11.5%).

**Table 1:** Baseline characteristics of gastric cancer patients according to EBV positivity

Variable	Total patients (*n* = 4587)	EBV-positive (*n* = 456) (9.9%)	EBV-negative (*n* = 4131) (90.1%)	*P* value
Age (mean ± SD)	61.1 (± 12.3)	61.2 (± 10.3)	61.1 (± 12.5)	0.780
≤ 60	2185 (47.6)	220 (48.2)	1965 (47.6)	0.783
> 60	2402 (52.4)	236 (51.8)	2166 (52.4)	
Sex				** < 0.001**
Male	3029 (66.0)	404 (88.6)	2625 (63.5)	
Female	1558 (34.0)	52 (11.4)	1506 (36.5)	
Smoking (*n* = 4,566)				** < 0.001**
Non-smoker	2230 (48.8)	137 (30.2)	2093 (50.9)	
Smoker	2336 (51.2)	316 (69.8)	2020 (49.1)	
Size (cm) (*n* = 4,341)	3.9	3.6	3.9	**0.030**
≤ 3	2213 (51.0)	236 (54.4)	1977 (50.6)	0.135
> 3	2128 (49.0)	198 (45.6)	1930 (49.4)	
Location (*n* = 4,580)				** < 0.001**
Upper	1021 (22.3)	236 (51.8)	785 (19.0)	
Middle	1055 (23.0)	131 (28.7)	924 (22.4)	
Low	2504 (54.7)	89 (19.5)	2415 (58.6)	
Histology				** < 0.001**
Differentiated	1893 (41.3)	123 (27.0)	1770 (42.9)	
Undifferentiated	1911 (41.7)	226 (49.5)	1685 (40.8)	
Mixed carcinoma	575 (12.5)	29 (6.4)	546 (13.2)	
Others	208 (4.5)	78 (17.1)	130 (3.1)	
Lauren classification (*n* = 4,346)				** < 0.001**
Intestinal	2356 (54.2)	215 (49.8)	2141 (54.7)	
Diffuse	1815 (41.8)	184 (42.6)	1631 (41.7)	
Mixed	130 (3.0)	10 (2.3)	120 (3.1)	
Indeterminate	45 (1.0)	23 (5.3)	22 (0.5)	
p53 mutation (*n* = 2,886)				** < 0.001**
Negative	2034 (70.5)	194 (87.8)	1840 (69.0)	
Positive	852 (29.5)	27 (12.2)	825 (31.0)	
MSI (*n* = 3,985)				** < 0.001**
MSS, MSI-L	3593 (90.2)	326 (99.4)	3,267 (89.3)	
MSI-H	392 (9.8)	2 (0.6)	390 (10.7)	
Cancer type				0.130
Early gastric cancer	2705 (59.0)	284 (62.3)	2421 (58.6)	
Advanced gastric cancer	1882 (41.0)	172 (37.7)	1710 (41.4)	
TNM staging				**0.004**
1	2821 (61.5)	316 (69.3)	2505 (60.6)	
2	685 (14.9)	52 (11.4)	633 (15.3)	
3	788 (17.2)	63 (13.8)	725 (17.6)	
4	293 (6.4)	25 (5.5)	268 (6.5)	
Lymphatic invasion (*n* = 4,345)				** < 0.001**
Absent	2836 (65.3)	328 (75.9)	2508 (64.1)	
Present	1509 (34.7)	104 (24.1)	1405 (35.9)	
Vascular invasion (*n* = 4,345)				**0.004**
Absent	3865 (89.0)	402 (93.1)	3463 (88.5)	
Present	480 (11.0)	30 (6.9)	450 (11.5)	
Treatment				0.138
Endoscopic submucosal Dissection	171 (3.7)	12 (2.6)	159 (3.8)	
Operation	3995 (87.1)	412 (90.4)	3583 (86.8)	
Chemotherapy	397 (8.7)	29 (6.4)	368 (8.9)	
Conservative care	24 (0.5)	3 (0.6)	21 (0.5)	

Bold indicates statistical signifcance

*EBV* Epstein Barr virus, *MSI* microsatellite instability, *MSS* microsatellite stable, *MSI-L* MSI low, *MSI-H* MSI-high

### Overall and gastric cancer-specific survival analyses according to EBV positivity

**Fig. 2:**
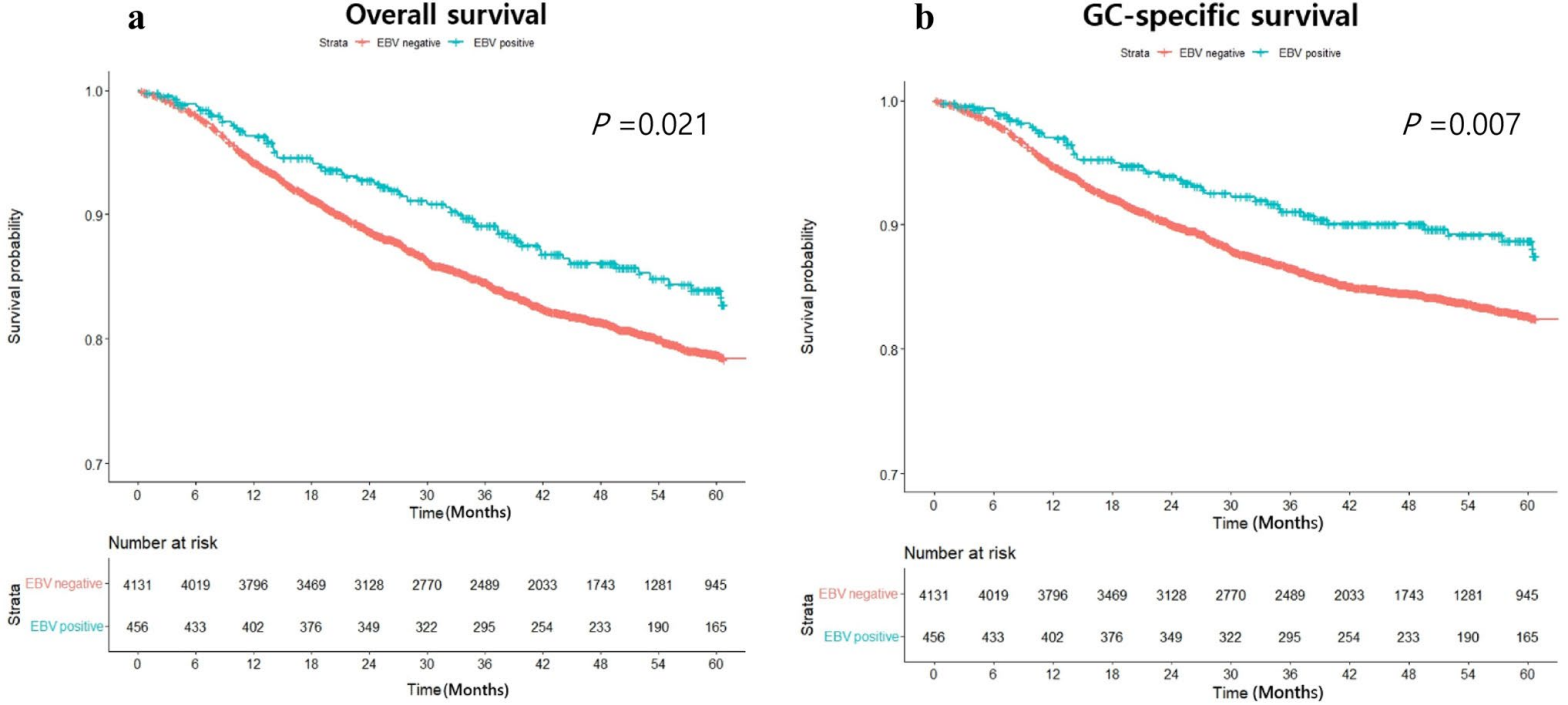
Comparisons of overall survival and gastric cancer-specific survival according to Epstein-Barrvirus (EBV) status. EBV positivity showed a positive effect significantly on both overall survival (**a**) and gastric cancer-specific survival (**b**)

Among the 4587 patients with GC, 766 patients died during the follow-up period, of which 626 died of GC and 140 of reasons other than GC. Overall survival analyses using the Kaplan–Meier method (Fig. 2a) indicated a significantly higher survival in the EBV-positive group than in the EBV-negative group. The 5-year cumulative survival rate in the EBV positive group vs. EBV negative group was 87.1% vs. 82.9% and it displayed a significant statistical *P* value validated using the log-rank test (*P* = 0.021). Moreover, the GC-specific survival analyses also indicated significantly higher survival rate in the EBV-positive group than in the EBV-negative group (Fig. 2b). Similar to the overall survival analyses, the 5-year cumulative survival rate was higher in the EBV-positive group than in the EBV-negative group (90.6% vs. 85.9%, *P* = 0.007).

### Univariate and multivariate analyses for survival according to EBV positivity

To evaluate the risk factors for overall survival and GC-specific survival, univariate and multivariate analyses were performed using the Cox proportional hazards regression. The results having statistical significance are summarized in Table 2. Univariate analyses included age, sex, smoking status, tumor size, tumor location, histology, Lauren classification type, TNM staging, and EBV–ISH positivity. Variables with a *P* < 0.05 in univariate analyses were used in multivariate analyses. For overall survival, univariate analyses revealed that age, sex, tumor size, tumor location, histology, Lauren classification type, cancer stage, and EBV positivity were associated with the mortality rate. In multivariate analyses, increasing age (hazard ratio [HR] = 1.037, *P* < 0.001) and tumor size (HR = 1.094, *P* < 0.001) were independent prognostic indicators. Tumor location in the lower third of the stomach (HR = 0.800, *P* = 0.033), cancer stage (stage 2 HR = 2.648, *P* < 0.001; stage 3 HR = 6.334, *P* < 0.001; stage 4 HR = 21.674, *P* < 0.001), and EBV positivity (HR = 0.671, *P* = 0.020) were also associated with mortality rate. The GC-specific survival study showed that univariate analyses revealed that age, tumor size, tumor location, histology, Lauren classification type, cancer stage, and EBV positivity were associated with the mortality rate. In multivariate analyses, increasing age (HR = 1.025, *P* < 0.001) and tumor size (HR = 1.109, *P* < 0.001) were independent prognostic indicators. Cancer stage (stage 2 HR = 4.761, *P* < 0.001; stage 3 HR = 13.286, *P* < 0.001; stage 4 HR = 42.528, *P* < 0.001) and EBV positivity (HR = 0.620, *P* = 0.022) were still associated with the GC-specific survival mortality rate.

**Table 2:** Univariate and multivariate analyses of risk factors for OS & GC-specific survival in study subjects

Variable	Overall Survival				Gastric cancer-specific survival			
	Univariate analyses HR [95% CI]	*P* value	Multivariate analyses HR [95% CI]	*P* value	Univariate analyses HR [95% CI]	*P* value	Multivariate analyses HR [95% CI]	*P* value
Age	1.030 (1.023–1.036)	** < 0.001**	1.037 (1.030–1.045)	** < 0.001**	1.018 (1.011–1.025)	** < 0.001**	1.025 (1.017–1.034)	** < 0.001**
Sex								
Male	Ref		Ref		Ref			
Female	0.813 (0.696–0.949)	**0.009**	0.761 (0.628–0.921)	0.005	0.917 (0.775–1.084)	0.310		
Smoking								
Non-smoker	Ref				Ref			
Smoker	0.970 (0.841–1.119)	0.676			0.943 (0.805–1.104)	0.467		
Size	1.254 (1.232–1.276)	** < 0.001**	1.094 (1.065–1.124)	** < 0.001**	1.295 (1.270–1.320)	** < 0.001**	1.109 (1.078–1.141)	** < 0.001**
Location								
Upper	Ref		Ref		Ref		Ref	
Middle	0.943 (0.784–1.133)	0.529	1.074 (0.849–1.358)	0.554	0.931 (0.763–1.136)	0.481	1.091 (0.834–1.427)	0.523
Low	0.500 (0.421–0.593)	** < 0.001**	0.800 (0.652–0.983)	**0.033**	0.437 (0.361–0.528)	** < 0.001**	0.841 (0.665–1.064)	0.149
Histology								
Differentiated	Ref		Ref		Ref		Ref	
Undifferentiated	1.987 (1.699–2.324)	** < 0.001**	1.178 (0.885–1.568)	0.261	2.538 (2.120–3.037)	** < 0.001**	1.178 (0.840–1.652)	0.342
Mixed	0.559 (0.405–0.774)	** < 0.001**	0.673 (0.449–1.008)	0.055	0.621 (0.428–0.901)	**0.012**	0.668 (0.415–1.072)	0.095
Others	1.476 (1.057–2.063)	**0.022**	1.034 (0.685–1.559)	0.875	1.644 (1.122–2.411)	**0.011**	1.042 (0.635–1.710)	0.871
Lauren								
Intestinal	Ref		Ref		Ref		Ref	
Diffuse	1.207 (1.019–1.430)	**0.030**	0.990 (0.755–1.297)	0.940	1.608 (1.322–1.954)	** < 0.001**	1.027 (0.752–1.403)	0.867
Mixed	1.042 (0.638–1.700)	0.870	1.221 (0.698–2.138)	0.484	1.141 (0.637–2.046)	0.657	1.108 (0.570–2.155)	0.762
Indeterminate	3.696 (2.021–6.759)	** < 0.001**	1.094 (1.065–1.124)	0.168	4.989 (2.637–9.439)	** < 0.001**	1.871 (0.871–4.017)	0.108
TNM staging								
Stage 1	Ref		Ref		Ref		Ref	
Stage 2	3.800 (2.935–4.920)	** < 0.001**	2.648 (2.017–3.476)	** < 0.001**	7.159 (5.005–10.239)	** < 0.001**	4.761 (3.277–6.916)	** < 0.001**
Stage 3	10.946 (8.864–13.518)	** < 0.001**	6.334 (4.918–8.157)	** < 0.001**	25.005 (18.385–34.009)	** < 0.001**	13.286 (9.400–18.777)	** < 0.001**
Stage 4	46.225 (37.005–57.743)	** < 0.001**	21.674 (14.706–31.945)	** < 0.001**	111.028 (81.071–152.055)	** < 0.001**	42.528 (26.903–67.226)	** < 0.001**
EBV-ISH positivity								
Negative	Ref		Ref		Ref		Ref	
Positive	0.733 (0.562–0.956)	**0.022**	0.671 (0.480–0.938)	**0.020**	0.666 (0.488–0.907)	**0.010**	0.620 (0.412–0.933)	**0.022**

Bold indicates statistical signifcance

*HR* Hazard ratio

### Subgroup analyses according to sex

As sex difference was expected, we divided the study groups according to sex in relation to EBV positivity. Baseline characteristics, depending on EBV positivity in both males and females, are summarized in Table 3. In males, the mean age of the EBV-positive group was 61.0 years, and 62.1 years in the EBV-negative group without significance. However, in females, the mean age of the EBV-positive group was 63.0 years, older than that in the EBV-negative group, i.e., 59.4 years old (*P* = 0.03). In both males and females, the predominant tumor location was in the upper third of the stomach in the EBV-positive group. Regarding the Lauren classification type, in males, the EBV-positive group (*n* = 404) displayed a significantly higher proportion of the diffuse-type than the EBV-negative group (*n* = 2625); however, this was not the case for females with the EBV-positive group (*n* = 52) than negative group (*n* = 1506). In terms of immune mechanism p53 mutation was lower in the EBV-positive group (11.7%) than negative group (36.6%) in males but no difference in female group (16.0% vs.21.1%). However, MSI did not show any difference depending on sex with GC by EBV positivity, suggesting that EBV-associated immune mechanism did not affect MSI in GC. Males in the EBV-positive group had a significantly higher proportion of EGC (62.4%) than in the EBV-negative group (56.7%); however, there was no significant difference among females (61.5% vs. 62.0%) (Table 3). Similarly, for the cancer stage, the male EBV-positive group had lower stages, whereas there was no significant difference in females. Regarding lymphatic and vascular invasion, males in the EBV-positive group showed significantly fewer cases of invasion, whereas females did not.

**Table 3:** Baseline characteristics of gastric cancer patients according to EBV positivity by sex

	Male (*n* = 3029)			Female (*n* = 1558)		
Variable	EBV-positive (*n* = 404) (13.3%)	EBV-negative (*n* = 2625) (86.7%)	*P* value	EBV-positive (*n* = 52) (3.3%)	EBV-negative (*n* = 1506) (96.7%)	*P* value
Age (mean ± SD)	61.0 (± 10.1)	62.1 (± 11.7)	0.054	63.0 (± 11.5)	59.4 (± 13.6)	**0.03**
≤ 60	200 (49.5)	1188 (45.3)	0.111	20 (38.5)	777 (51.6)	0.063
> 60	204 (50.5)	1437 (54.7)		32 (61.5)	729 (48.4)	
Smoking	(*n* = 3015)		**0.046**	(*n* = 1551)		0.620
Non-Smoker	90 (22.4)	710 (27.2)		47 (90.4)	1383 (92.3)	
Smoker	311 (77.6)	1904 (72.8)		5 (9.6)	116 (7.7)	
Size (cm)	(*n* = 2864)			(*n* = 1477)		
	3.7 (± 2.7)	4.0 (± 3.0)	0.065	3.1 (± 1.9)	3.8 (± 3.1)	**0.018**
≤ 3	211 (54.9)	1223 (49.3)	**0.041**	25 (50.0)	754 (52.8)	0.693
> 3	173 (45.1)	1257 (50.7)		25 (50.0)	673 (47.2)	
Location	(*n* = 3026)		** < 0.001**	(*n* = 1554)		** < 0.001**
Upper	208 (51.5)	502 (19.1)		28 (53.8)	283 (18.8)	
Middle	115 (28.5)	512 (19.5)		16 (30.8)	412 (27.4)	
Low	81 (20.0)	1608 (61.3)		8 (15.4)	807 (53.7)	
Histology			** < 0.001**			** < 0.001**
Differentiated	112 (27.7)	1334 (50.8)		11 (21.2)	436 (29.0)	
Undifferentiated	202 (50.1)	902 (34.4)		24 (46.2)	783 (52.0)	
Mixed carcinoma	24 (5.9)	286 (10.9)		5 (9.6)	260 (17.3)	
Others	66 (16.3)	103 (3.9)		12 (23.0)	27 (1.7)	
Lauren classification	(*n* = 2867)		** < 0.001**	(*n* = 1479)		** < 0.001**
Intestinal	197 (51.6)	1584 (63.7)		18 (36.0)	557 (39.0)	
Diffuse	157 (41.1)	818 (32.9)		27 (54.0)	813 (56.9)	
Mixed	7 (1.8)	64 (2.6)		3 (6.0)	56 (3.9)	
Indeterminate	21 (5.5)	19 (0.8)		2 (4.0)	3 (0.2)	
p53 mutation	(*n* = 1894)		** < 0.001**	(*n* = 992)		0.537
Negative	173 (88.3)	1077 (63.4)		21 (84.0)	763 (78.9)	
Positive	23 (11.7)	621 (36.6)		4 (16.0)	204 (21.1)	
MSI	(*n* = 2635)		** < 0.001**	(*n* = 1350)		**0.024**
MSS, MSI-L	291 (99.3)	2120 (90.5)		35 (100.0)	1147 (87.2)	
MSI-H	2 (0.7)	222 (9.5)		0 (0.0)	168 (12.8)	
Cancer type			**0.032**			0.944
Early gastric cancer	252 (62.4)	1489 (56.7)		32 (61.5)	934 (62.0)	
Advanced gastric cancer	152 (37.6)	1136 (43.3)		20 (38.5)	572 (38.0)	
TNM staging			**0.001**			0.528
1	280 (69.3)	1555 (59.3)		36 (69.2)	950 (63.1)	
2	44 (10.9)	416 (15.8)		8 (15.4)	217 (14.4)	
3	56 (13.9)	485 (18.5)		7 (13.5)	240 (15.9)	
4	24 (5.9)	169 (6.4)		1 (1.9)	99 (6.6)	
Lymphatic invasion	(*n* = 2866)		** < 0.001**	(*n* = 1479)		0.399
Absent	291 (76.2)	1531 (61.6)		37 (74.0)	977 (68.4)	
Present	91 (23.8)	953 (38.4)		13 (26.0)	452 (31.6)	
Vascular invasion	(*n* = 2866)		**0.001**	(*n* = 1479)		0.267
Absent	354 (92.7)	2154 (86.7)		48 (96.0)	1309 (91.6)	
Present	28 (7.3)	330 (13.3)		2 (4.0)	120 (8.4)	
Treatment			0.084			0.685
Endoscopic submucosal dissection	11 (2.7)	118 (4.5)		1 (1.9)	41 (2.7)	
Operation	363 (89.9)	2242 (85.4)		49 (94.2)	1342 (89.1)	
Chemotherapy	27 (6.7)	250 (9.5)		2 (3.8)	117 (7.8)	
Conservative care	3 (0.7)	15 (0.6)		0 (0.0)	6 (0.4)	

Bold indicates statistical signifcance

**Fig. 3:**
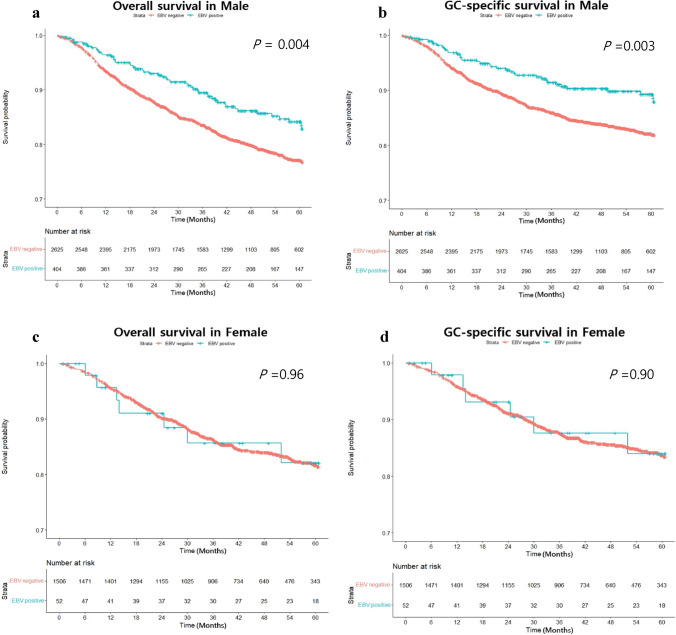
Comparisons of overall survival and gastric cancer-specific survival according to Epstein-Barr virus (EBV) status in male and female. EBV positivity showed a positive effect significantly on both overall survival (**a**) and gastric cancer-specific survival (**b**) in male but no significant difference in female neither overall survival (**c**) nor gastric cancer-specific survival (**d**)

Overall survival and GC-specific survival analyses using the Kaplan–Meier method revealed significantly higher survival rates for both overall survival and GC-specific survival in males in the EBV-positive group than those in the EBV-negative group. In overall survival analyses, 5-year cumulative survival rates in EBV-positive group and EBV-negative groups in males were 87.1% and 81.4%, respectively (*P* = 0.004) (Fig. 3a). In GC-specific survival analyses, 5-year cumulative survival rates in the EBV-positive group and EBV-negative groups in male were 90.8% and 85.3%, respectively (Fig. 3b) (*P* = 0.003). However, in females, no statistically significant difference was observed between the EBV-positive and EBV-negative group. (OS: *P* = 0.96, GCSS: *P* = 0.90) (Fig. 3c, Fig. 3d).

Next, we evaluated the risk factors for overall survival and GC-specific survival in both male and female sub-analyses by univariate and multivariate analyses, and the results are summarized in Table 4. Univariate analyses included age, smoking status, tumor size, tumor location, histology, Lauren classification type, TNM staging, and EBV–ISH positivity. In multivariate analyses, GC-specific survival revealed that EBV positivity was an independent good prognostic factor for mortality in males. (GCSS: HR = 0.517, *P* = 0.005) (Table 4). In contrast, EBV positivity was not a prognostic factor in females (Table 4).

**Table 4:** Univariate and multivariate analyses of risk factors for GC-specific survival according to sex

Variable	Male				Female			
	Univariate analyses HR [95% CI]	*P* value	Multivariate analyses HR [95% CI]	*P* value	Univariate analyses HR [95% CI]	*P* value	Multivariate analyses HR [95% CI]	*P* value
Age	1.032 (1.023–1.041)	< 0.001	1.042 (1.031–1.054)	< 0.001	1.000 (0.989–1.010)	0.932		
Smoking								
Non-smoker	Ref		Ref		Ref			
Smoker	0.747 (0.609–0.917)	**0.005**	0.996 (0.772–1.283)	0.973	1.531 (0.991–2.365)	0.055		
Size	1.297 (1.266–1.328)	** < 0.001**	1.102 (1.063–1.142)	** < 0.001**	1.290 (1.249–1.333)	** < 0.001**	1.134 (1.079–1.191)	** < 0.001**
Location								
Upper	Ref		Ref		Ref		Ref	
Middle	1.141 (0.887–1.467)	0.304	1.129 (0.817–1.560)	0.463	0.637 (0.460–0.883)	**0.007**	1.017 (0.614–1.683)	0.949
Low	0.578 (0.460–0.728)	** < 0.001**	0.847 (0.642–1.118)	0.242	0.242 (0.171–0.343)	** < 0.001**	0.754 (0.481–1.182)	0.218
Histology								
Differentiated	Ref		Ref		Ref		Ref	
Undifferentiated	2.869 (2.319–3.549)	** < 0.001**	1.260 (0.847–1.876)	0.254	1.992 (1.411–2.811)	** < 0.001**	0.685 (0.431–1.088)	0.109
Mixed	0.637 (0.389–1.044)	0.073	0.671 (0.368–1.222)	0.192	0.567 (0.314–1.022)	0.059	0.377 (0.195–0.730)	**0.004**
Others	1.879 (1.249–2.827)	**0.002**	1.268 (0.752–2.139)	0.373	0.745 (0.231–2.404)	0.622	0.377 (0.090–1.586)	0.183
Lauren								
Intestinal	Ref		Ref		Ref			
Diffuse	1.791 (1.415–2.267)	** < 0.001**	1.172 (0.812–1.692)	0.398	1.396 (0.968–2.013)	0.074		
Mixed	1.606 (0.819–3.150)	0.168	1.684 (0.775–3.657)	0.188	0.603 (0.187–1.945)	0.397		
Indeterminate	4.794 (2.440–9.419)	** < 0.001**	1.648 (0.737–3.684)	0.224	4.819 (0.662–35.085)	0.121		
TNM staging								
Stage 1	Ref		Ref		Ref		Ref	
Stage 2	7.425 (4.816–11.448)	** < 0.001**	4.791 (3.045–7.539)	** < 0.001**	6.317 (3.337–11.957)	** < 0.001**	4.005 (2.050–7.827)	** < 0.001**
Stage 3	25.175 (17.304–36.627)	** < 0.001**	13.084 (8.564–19.989)	** < 0.001**	23.507 (13.722–40.272)	** < 0.001**	11.817 (6.447–21.659)	** < 0.001**
Stage 4	103.874 (70.561–152.914)	** < 0.001**	38.129 (20.594–70.595)	** < 0.001**	120.019 (69.874–206.149)	** < 0.001**	58.179 (28.131–120.322)	** < 0.001**
EBV-ISH positivity								
Negative	Ref		Ref		Ref			
Positive	0.599 (0.428–0.840)	**0.003**	0.517 (0.327–0.816)	**0.005**	0.949 (0.421–2.138)	0.949		

Bold indicates statistical signifcance

## Discussion

In our study, the EBVaGC group showed a higher proportion of males (*P* < 0.001), with tumors predominantly in the proximal stomach (*P* < 0.001), a higher proportion of undifferentiated cancer (*P* < 0.001), and a lower cancer stage (*P* = 0.004) than the EBV-negative group. For lymphatic and vascular invasion, EBV-positive group had significantly fewer cases of lymphatic and vascular invasion (lymphatic; *P* < 0.001, vascular; *P* = 0.004). Furthermore, the EBVaGC group displayed statistically significant survival advantages over the EBV-negative cancer group in terms of both overall and GC-specific survival. However, this effect was evident only in males.

EBVaGC accounts for approximately 10% of all GCs, although there are differences in each study. For instance, Kim et al. have reported an incidence of 6.27% (21 of 335 individuals) of EBVaGC [12]. In Tokunaga's study, 67 cases (6.9%) of 970 individuals were EBV-positive [33]. In addition, Lee's meta-analyses revealed an incidence of 8.8%, with 857 of 9,738 individuals having EBVaGC [22]. In a study by van Beek [14], of 566 individuals, 41 (7.2%) were EBV positive, of which 38 (92.7%) were male [14]. In our study, 456 patients with GC (9.9%) among 4,587 GC patients were EBV positive and the number of males was 404 (88.6%), similar to that reported in van Beek’s study. In addition, van Beek reported that the undifferentiated type was significantly higher in EBVaGC (76.2%), and the tumor location predominantly appeared in the proximal region of the stomach in 82.9% of patients [14]. Consistent with this, our study displayed a predominant presence in the proximal stomach at 51.8% and a higher proportion of undifferentiated tumor type (49.5%). Despite these unfavorable conditions for the prognosis of GC, we found a negative correlation between EBV positivity and lymphovascular invasion in GC, as previously demonstrated by Lee et al. [22], and Park et al. [34], and others [35, 36]. In our study, EBV positivity in GC was an independent factor associated with favorable survival outcomes. EBVaGCs are characterized by dense lymphoid cell infiltration of the gastric stroma, leading to an active host cell immune response [8, 37, 38]. This robust immune activity triggers an inflammatory response causing the fusion of cancer cells, exhibiting a “lace pattern” [39]. These cancer cells lack tubule formation and are histologically classified as undifferentiated type for these reasons. Although undifferentiated types of cancer are commonly associated with poor prognosis in GC, the active host cell immune response serves as a protective factor in EBVaGC, such as the inhibition of lymph node metastasis or vascular invasion, thereby yielding characteristically favorable prognoses despite the undifferentiated histology [40]. To date, there have been no reports of sex differences in EBVaGC. This could be attributed to the higher prevalence of GC in males than in females worldwide, and no research group has performed a subgroup analysis of EBVaGC regarding sex. However, GC showed a sex-based difference. For instance, younger patients with GC are more likely to be females, have the diffuse- and undifferentiated types of GC, and present with AGC. In contrast, older patients with GC are more likely to be males, have intestinal-type GC, and present with simultaneous tumors [41]. Intestinal-type GC was significantly less frequent in premenopausal females (19.0%) and postmenopausal females aged < 10 years (30.4%) and 10 to 19 years old (44.1%) after menopause compared to males (61%) (all *P* value < 0.05) [42]. However, this significant difference in the proportion of intestinal-type GC was not observed between males and females ≥ 20 years after menopause (60.6 vs. 61%, *P* = 0.518) [42]. Changes in the proportions of intestinal and diffuse-type cancers suggest that estrogen could have a protective effect on intestinal-type GC. EBV was the first virus to be found in human tumors and has been implicated in several malignancies such as Burkitt's lymphoma, non-Hodgkin's lymphoma, Hodgkin's lymphoma, and nasopharyngeal carcinoma, most of which are intricately related to immune cells. Estrogen and testosterone have direct effects on immune cells. β-estradiol stimulates dendritic cells to secrete interleukin (IL)-12 and interferon (IFN)-γ, which in turn activate the secretion of proinflammatory cytokines. In addition, β-estradiol extends the survival of B lymphocytes, activates polyclonal B lymphocytes, increases intestinal permeability, and create a pro-inflammatory environment. In males, testosterone inhibits the proliferation of T lymphocytes and interferes with the Toll-like receptor (TLR) mechanism, which is different from the effect of β-estradiol effect on B lymphocytes. Our findings revealed that the EBV positivity was a favorable prognostic factor in males but not in females. Females generally show stronger immune functions not only in cancer, but also in viral and microbial infections than males. A greater number of differentially activated immune processes have been observed in GC in females [31, 32]. In addition, a study investigating tumor-associated neutrophils (TANs), a part of the tumor immune microenvironment (TIME), reported that an increase in the number of TANs correlated with a better prognosis only in females with GC [43]. Sex differences in the TIME had been observed in various types of cancer. Ye Y et al. summarized meta-analysis regarding melanoma and lung cancer, the most common types of cancers, in the largest number of immune checkpoint blockade (ICB) clinical trials [44]. There was a higher tumor mutation burden (TMB) and PD-L1 expression in male patients with melanoma than in females [44]. In contrast, female displayed higher activity of CD4 + and CD8 + cells than in males with lung squamous cell cancer [44]. These differences affected overall survival with ICB therapy in various cancers according to sex [44]. However to date, studies on sex differences about TIME has shown inconsistent results and there is lack of studies about EBV-related cancers such as Burkitt’s lymphoma, Hodgkin’s lymphoma, and nasopharyngeal cancer. Although the underlying mechanism is unclear so far, the reason why EBV positivity had a more pronounced impact on prognosis in males in our study could be that females already have an active immune response against GC, resulting in EBVaGC being 4.03 times higher in males than females. In addition, the immunogenicity of EBVaGC did not yield significant differences in prognosis compared with that of GC. These results could be useful for other EBV-associated tumors. Thus, further research is required to assess the sex differences in EBV-associated tumors including GC with respect to EBV infection.

This study had certain limitations. First, although the Kaplan–Meier curve suggested differences in the impact of EBV positivity between males and females, fewer female patients with EBVaGC than male patients could have influenced the results. This disparity could potentially affect the observed survival rate differences between the female EBVaGC and GC groups, resulting in higher *P* values. However, we believe that this is further evidence of the relatively stronger immune mechanisms in females. Second, this study was conducted at a single institution within a single country, deficient in national differences, or was a multicenter study. Thus, a meta-analysis of this topic, including our results, is required in the future. In addition, considering that our medical institution is a tertiary institution in which patients with relatively severe conditions are treated, there may have been a selection bias in patient recruitment. Despite these limitations, our study has several strengths. The number of GC cases was rather large, and the prospective survival rate was provided by the surgical and medical cohorts from 2003.

In conclusion, patients with EBVaGC displayed better prognosis despite its association with proximal location and poorly differentiated histology only in males, suggesting that EBV infection causes GC using sex-specific immune mechanisms.
